# Supplementary material for: Management capacity of primary healthcare facilities in low- and middle-income countries: A scoping review
Source: PLOS Glob Public Health. 2025 Jul 23;5(7):e0004445. doi: 10.1371/journal.pgph.0004445 (PMC12286403; doi:10.1371/journal.pgph.0004445)
Supplement: S1 Data — This file contains the search terms that were used to carry out the search in PubMed, date each of the searches were conducted and the results. The search terms are structured per the Population Concept Context (PCC) framework as discussed in the manuscript. (DOCX) [file pgph.0004445.s002.docx]

**PUBMED Search Strategy**

| **#** | **Query** | **Date Searched** | **Results** |
| --- | --- | --- | --- |
| **S1** | ((("manager" OR "administrator" OR "supervisor" OR "coordinator" OR "head" OR "lead" OR "overseer" OR "director" OR "controller" OR "executive" OR "manag*" OR "administrat*" OR "supervis*" OR "coordinat*" OR "overs*" OR "direct*" OR "control*" OR "execut*" OR "operat*" OR "operat*" OR "steer*" OR "running" OR "chair*" OR "officer" OR "in-charge" OR "coordinat*" OR "chief" OR "superintendent")) AND ((("managerial capacity"[tiab:~2] OR "leadership capacity"[tiab:~2] OR "administrative capacity"[tiab:~2] OR "governance capacity"[tiab:~2]) OR ("manag*" AND "Capacit*") OR ("lead*" AND "capacit*") OR ("govern*" AND "capacit*") OR ("administrat*" AND "capacit*")) OR ("organi*" AND "capacit*") OR "capacity building"[MeSH Terms] OR ("manag*" AND "competenc*") OR ("facility manag*"))) | 24/10/2024 | 242,040 |
| **S2** | (("primary health care"[tiab] OR "primary care"[tiab] OR "primary healthcare"[tiab] OR "Primary health" OR "community health" OR "community health care"[tiab] OR "community healthcare"[tiab] OR "community care" OR "Community health cent*"[tiab] OR "community health clinics"[tiab] OR "health cent*"[tiab] OR "health cent*"[tiab] OR "health clinics"[tiab] OR "district health service*"[tiab] OR "district health" OR "preventive health service*"[tiab] OR "primary level" OR "primary-level" OR "frontline health" OR "frontline healthcare" OR "rural health*" OR "general practice" OR "family practice" OR "family medicine") AND (("Primary Health Care"[MeSH Terms]) OR ("Community Health Service*"[MeSH Terms]))) | 24/10/2024 | 183,508 |
| **S3** | ((Afghanistan[TIAB] OR Albania[TIAB] OR Algeria[TIAB] OR American Samoa[TIAB] OR Angola[TIAB] OR Antigua[TIAB] OR Barbuda[TIAB] OR Argentina[TIAB] OR Armenia[TIAB] OR Azerbaijan[TIAB] OR Bangladesh[TIAB] OR Belarus[TIAB] OR Byelarus[TIAB] OR Byelorussia[TIAB] OR Belorussia[TIAB] OR Belize[TIAB] OR Benin[TIAB] OR Bhutan[TIAB] OR Bolivia[TIAB] OR Bosnia[TIAB] OR Herzegovina[TIAB] OR Hercegovina[TIAB] OR BosniaHerzegovina[TIAB] OR Bosnia-Hercegovina[TIAB] OR Botswana[TIAB] OR Brazil[TIAB] OR Brasil[TIAB] OR Bulgaria[TIAB] OR Burkina[TIAB] OR Upper Volta[TIAB] OR Burundi[TIAB] OR Urundi[TIAB] OR Cambodia[TIAB] OR Republic of Kampuchea[TIAB] OR Cameroon[TIAB] OR Cameroons[TIAB] OR Cape Verde[TIAB] OR Central African Republic[TIAB] OR Chad[TIAB] OR Chile[TIAB] OR China[TIAB] OR Colombia[TIAB] OR Comoros[TIAB] OR Comoro Islands[TIAB] OR Comores[TIAB] OR Congo[TIAB] OR DRC[TIAB] OR Zaire[TIAB] OR Costa Rica[TIAB] OR Cote d'Ivoire[TIAB] OR Ivory Coast[TIAB] OR Cuba[TIAB] OR Djibouti[TIAB] OR Obock[TIAB] OR French Somaliland[TIAB] OR Dominica[TIAB] OR Dominican Republic[TIAB] OR Ecuador[TIAB] OR Egypt[TIAB] OR United Arab Republic[TIAB] OR El Salvador[TIAB] OR Eritrea[TIAB] OR Ethiopia[TIAB] OR Fiji[TIAB] OR Gabon[TIAB] OR Gabonese Republic[TIAB] OR Gambia[TIAB] OR Georgia[TIAB] OR Ghana[TIAB] OR Gold Coast[TIAB] OR Grenada[TIAB] OR Guatemala[TIAB] OR Guinea[TIAB] OR GuineaBissau[TIAB] OR Guiana[TIAB] OR Guyana[TIAB] OR Haiti[TIAB] OR Honduras[TIAB] OR India[TIAB] OR Indonesia[TIAB] OR Iran[TIAB] OR Iraq[TIAB] OR Jamaica[TIAB] OR Jordan[TIAB] OR Kazakhstan[TIAB] OR Kenya[TIAB] OR Kiribati[TIAB] OR Republic of Korea[TIAB] OR North Korea[TIAB] OR DPRK[TIAB] OR Kosovo[TIAB] OR Kyrgyzstan[TIAB] OR Kirghizstan[TIAB] OR Kirgizstan[TIAB] OR Kirghizia[TIAB] OR Kirgizia[TIAB] OR Kyrgyz[TIAB] OR Kirghiz[TIAB] OR Kyrgyz Republic[TIAB] OR Lao[TIAB] OR Laos[TIAB] OR Latvia[TIAB] OR Lebanon[TIAB] OR Lesotho[TIAB] OR Basutoland[TIAB] OR Liberia[TIAB] OR Libya[TIAB] OR Lithuania[TIAB] OR Macedonia[TIAB] OR Madagascar[TIAB] OR Malagasy Republic[TIAB] OR Malawi[TIAB] OR Nyasaland[TIAB] OR Malaysia[TIAB] OR Malaya[TIAB] OR Malay[TIAB] OR Maldives[TIAB] OR Mali[TIAB] OR Marshall Islands[TIAB] OR Mauritania[TIAB] OR Mauritius[TIAB] OR Mayotte[TIAB] OR Mexico[TIAB] OR Micronesia[TIAB] OR Moldova[TIAB] OR Moldovia[TIAB] OR Mongolia[TIAB] OR Montenegro[TIAB] OR Morocco[TIAB] OR Mozambique[TIAB] OR Myanmar[TIAB] OR Burma[TIAB] OR Namibia[TIAB] OR Nepal[TIAB] OR Nicaragua[TIAB] OR Niger[TIAB] OR Nigeria[TIAB] OR Pakistan[TIAB] OR Palau[TIAB] OR Palestine[TIAB] OR Panama[TIAB] OR Papua New Guinea[TIAB] OR Paraguay[TIAB] OR Peru[TIAB] OR Philippines[TIAB] OR Romania[TIAB] OR Rumania[TIAB] OR Roumania[TIAB] OR Russia[TIAB] OR Russian Federation[TIAB] OR USSR[TIAB] OR Soviet Union[TIAB] OR Union of Soviet Socialist Republics[TIAB] OR Rwanda[TIAB] OR RuandaUrundi[TIAB] OR Samoa[TIAB] OR Samoan Islands[TIAB] OR Sao Tome[TIAB] OR Principe[TIAB] OR Senegal[TIAB] OR Serbia[TIAB] OR Montenegro[TIAB] OR Yugoslavia[TIAB] OR Seychelles[TIAB] OR Sierra Leone[TIAB] OR Solomon Islands[TIAB] OR Somalia[TIAB] OR South Africa[TIAB] OR Sri Lanka[TIAB] OR Ceylon[TIAB] OR Saint Kitts[TIAB] OR St Kitts[TIAB] OR Saint Christopher Island[TIAB] OR Nevis[TIAB] OR Saint Lucia[TIAB] OR St Lucia[TIAB] OR Saint Vincent[TIAB] OR St Vincent[TIAB] OR Grenadines[TIAB] OR Sudan[TIAB] OR Suriname[TIAB] OR Surinam[TIAB] OR Swaziland[TIAB] OR Syria[TIAB] OR Syrian Arab Republic[TIAB] OR Tajikistan[TIAB] OR Tadzhikistan[TIAB] OR Tadjikistan[TIAB] OR Tanzania[TIAB] OR Thailand[TIAB] OR Timor-Leste[TIAB] OR East Timor[TIAB] OR Togo[TIAB] OR Togolese Republic[TIAB] OR Tonga[TIAB] OR Tunisia[TIAB] OR Turkey[TIAB] OR Turkmenistan[TIAB] OR Turkmenia[TIAB] OR Tuvalu[TIAB] OR Uganda[TIAB] OR Ukraine[TIAB] OR Uruguay[TIAB] OR Uzbekistan[TIAB] OR Vanuatu[TIAB] OR New Hebrides[TIAB] OR Venezuela[TIAB] OR Vietnam[TIAB] OR Viet Nam[TIAB] OR West Bank[TIAB] OR Gaza[TIAB] OR Yemen[TIAB] OR Zambia[TIAB] OR Zimbabwe[TIAB] OR Rhodesia[TIAB]) OR (LIC[TIAB] OR LICs[TIAB] OR MIC[TIAB] OR MICs[TIAB] OR LMIC[TIAB] OR LMICs[TIAB] OR LAMIC[TIAB] OR LAMICs[TIAB] OR LAMI countr*[TIAB])) | 24/10/2024 | 1,693,663 |
| **S4** | **S1 AND S2 AND S3** | **24/10/2024** | **1356** |
