# Supplementary material for: Management capacity of primary healthcare facilities in low- and middle-income countries: A scoping review
Source: PLOS Glob Public Health. 2025 Jul 23;5(7):e0004445. doi: 10.1371/journal.pgph.0004445 (PMC12286403; doi:10.1371/journal.pgph.0004445)
Supplement: S4 Data — This file contains the search terms that were used to carry out the search in Google Scholar, when the search was conducted and the results of this search. (DOCX) [file pgph.0004445.s005.docx]

**Google Scholar Search**

| **#** | **Search Terms** | **Date Searched** | **Results** |
| --- | --- | --- | --- |
| **S1** | "managerial capacity" OR "leadership capacity" OR "administrative capacity" OR "governance capacity" OR "management competence" OR "capacity building" AND ("primary health care" OR "community health" OR "health clinics" OR "district health services" OR "family medicine") | 24/10/2024 | 19,800  Picked 1^st^ 200 (Explanation in manuscript) |
